# *Mvda* is required for zebrafish early development

**DOI:** 10.1186/s40659-021-00341-7

**Published:** 2021-05-29

**Authors:** Wenghong Wong, Yike Huang, Zhuanbin Wu, Yu Kong, Jing Luan, Qiaoan Zhang, Jiewen Pan, Kexiang Yan, Zhenghua Zhang

**Affiliations:** 1 Department of Dermatology, Huashan Hospital, Shanghai Medical College of Fudan University, Shanghai, China; 2 Department of Dermatology, Xiamen Chang Gung Hospital, Xiamen, China; 3 Shanghai Model Organisms Center Inc, Shanghai, China; 4 Institute of Neuroscience, Chinese Academy of Science, Shanghai, China

**Keywords:** *Mvda*, Mevalonate pathway, Zebrafish, Epidermis, Angiogenesis

## Abstract

**Background:**

The *MVD* gene mutations are identified in porokeratosis, which is considered a skin-specific autoinflammatory keratinization disease. However, the biological function of *MVD* gene remains largely unknown. Therefore, we analyzed the function of *mvda* gene, orthologous to the human *MVD* gene, in developing zebrafish.

**Methods:**

Morpholino antisense oligonucleotide technique was used to generate *mvda* loss-of-function phenotypes. Knockdown of *mvda* was confirmed by RT-PCR and Sanger sequencing. Scanning and transmission electron microscopy were performed to analyze the morphology of the epidermis. Angiogenesis study was presented using the *Tg(fli1a:EGFP)*^*y1*^ transgenic strain. In addition, acridine orange staining was used to examine the apoptotic cells in vivo*.*

**Results:**

As expected, the *mvda* morphants showed abnormal morphology of the epidermis. Moreover, we observed ectopic sprouts in trunk angiogenesis and impaired formation of the caudal vein plexus in the *mvda*-deficient zebrafish. Besides, increased apoptosis was found throughout the tail, heart, and eyes in *mvda* zebrafish morphants.

**Conclusions:**

These findings indicated the essential role of *mvda* in the early development of zebrafish. This was the first in vivo knockdown study of the zebrafish *mvda* gene, which might offer insight into the biological function of the human *MVD* gene.

**Supplementary Information:**

The online version contains supplementary material available at 10.1186/s40659-021-00341-7.

## Background

Mevalonate diphosphate decarboxylase (MVD) is a key enzyme that catalyzes mevalonate-5-pyrophosphate into isopentenyl-5-pyrophosphate in the mevalonate pathway. The final products of the mevalonate pathway include cholesterol, steroid hormones, and non-steroid isoprenoids, which are necessary for cell survival. Mutations in the gene encoding MVD are the commonest cause in a cohort of Chinese patients with porokeratosis (PK, MIM 175800), which is considered a skin-specific autoinflammatory keratinization disease. PK exhibits an autosomal dominant mode of inheritance, featured by multiple superficial keratotic lesions with a slightly raised keratotic border [1–5]. However, the biological function of *MVD* gene remains largely unknown.

The development and function of zebrafish organs are strikingly similar to those of humans, and the ease of creating mutant or transgenic fish has facilitated the generation of disease models. Zebrafish have become a popular organism for the study of vertebrate gene function. The virtually transparent embryos of this species, and the ability to accelerate genetic studies by gene knockdown or overexpression, have led to the widespread use of zebrafish in the detailed investigation of vertebrate gene function and increasingly, the study of human genetic disease [6, 7]. Here we employed zebrafish model and morpholino oligonucleotides (MO) knockdown technology to characterize the biological function of zebrafish *mevalonate *(*diphosphate*)* decarboxylase a *(*mvda*) gene, orthologous to the human *MVD* gene, during early development in zebrafish. Zebrafish *mvda* gene is 65.6% homologous to that of the human *MVD* gene and 67.5% conserved at the protein level. Knockdown of gene function by MO is the most familiar genetic approach to study genes of interest in zebrafish [8]. By specifically interfering with the mRNA transcripts, MO avoids the genetic compensation response which is induced by deleterious mutations but not gene MO knockdowns. [9, 10].

Here, we report that *mvda* is expressed during early zebrafish development. Inhibition of *mvda* expression using MO blocks zebrafish early embryogenesis, leading to dramatic developmental defects. Morphant embryos display epidermis and vascular defects, as well as increased apoptosis throughout the tail, heart, and eyes. These in vivo results suggest that *mvda* is a key factor during the early steps of zebrafish development. Our findings provided the first clue to understanding the function of *MVD*.

## Results

### *mvda* deficiency impairs regular development of zebrafish

**Fig. 1:**
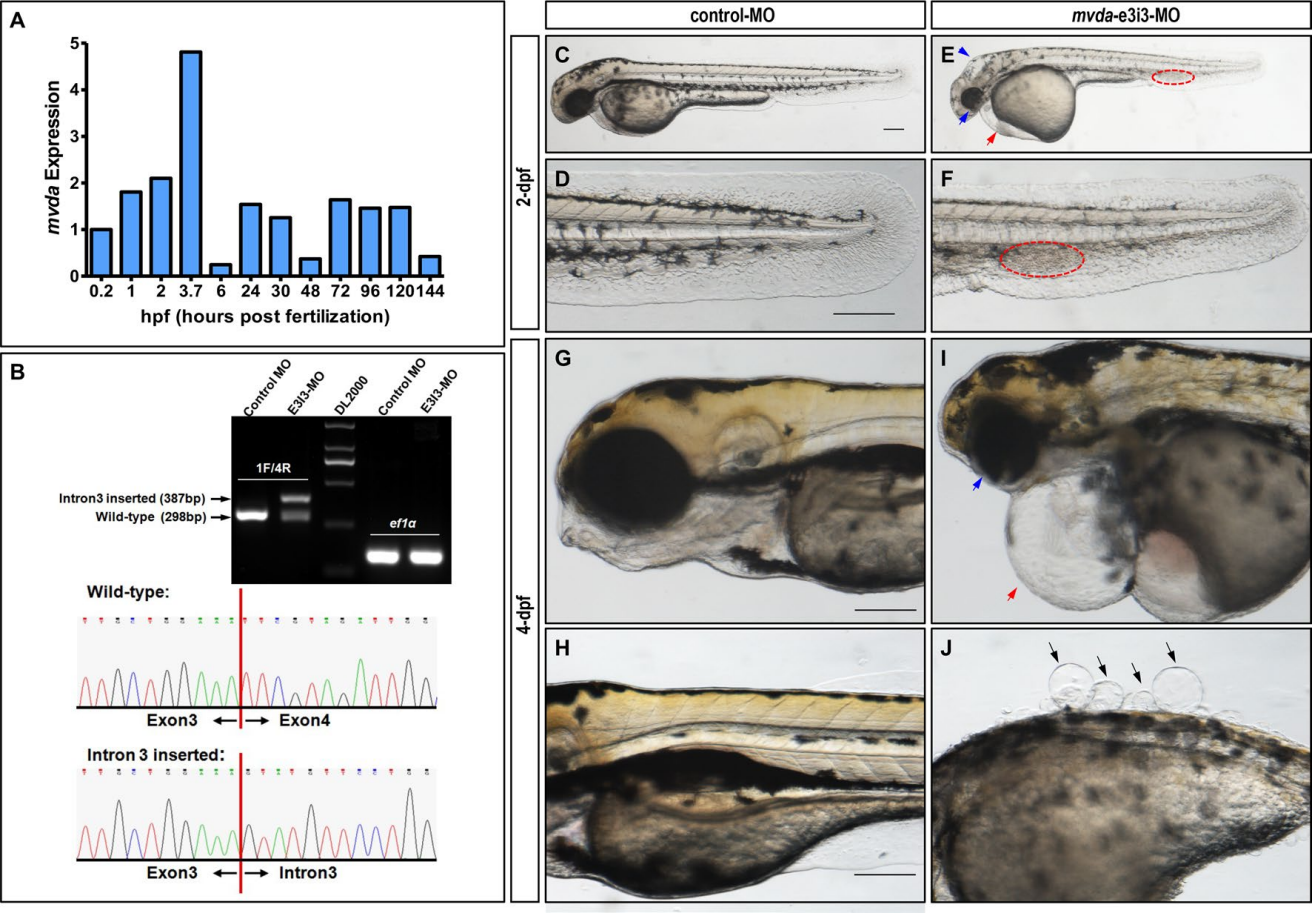
Aberrant *mvda* function causes developmental defects in zebrafish. **A** Quantitative real-time PCR (qRT-PCR) for twelve embryo development stages (0.2 hpf, 1 hpf, 2 hpf, 3.7 hpf, 6 hpf, 24 hpf, 30 hpf, 48 hpf, 72 hpf, 96 hpf, 120 hpf, and 144 hpf) demonstrates different expression patterns of *mvda* during embryonic development. **B** Effectiveness of *mvda* knockdown was confirmed by RT-PCR and sanger sequencing. The zebrafish *mvda* gene was targeted by specific morpholino antisense to prevent the proper splicing of exon 3 (E3I3-MO). Primers spanning *mvda* exon 1 (forward) and exon 4 (reverse) interrogate the presence of wild type (non-mutant) transcripts or those in which intron 3 has been inserted. RT-PCR of *mvda* transcript from control-MO and E3I3-MO injected embryos at 2-dpf, demonstrating insertion of intron 3. Sanger sequencing of both the wild-type band and the intron 3-inserted band validating the wild-type sequence and the intron 3-inserted sequence. **C**–**J** Gross morphology at 2-dpf and 4-dpf. Compared with the control group, knockdown of *mvda* presented hydrocephaly (**E**, blue arrowhead), eye defects (**E**, **I**, blue arrow), pericardial oedema (**E**, **I**, red arrow), blood accumulation in the caudal vein (**E**, **F**, red circled area), and skin defects (**J**, black arrows). Heartbeat and circulation in the caudal vein were visible in the control fish but were abnormal in *mvda* morphants (Additional file 2: Video S1, Additional file 3: Video S2). hpf, hours post fertilization; dpf, days post fertilization. Scale bars = 100 µm

The expression patterns of *mvda* gene were different in the embryonic development stages of zebrafish (Fig. 1A). *mvda* knockdown was generated by inserting the intron 3 sequence into its transcript, and the effectiveness was confirmed by reverse transcription-PCR (RT-PCR) and Sanger sequencing (Fig. 1B). The gross morphological phenotypes of *mvda* morphants at 2 days post fertilization (dpf) and 4-dpf were depicted (Fig. 1C–J), including skin defects, pericardial oedema, blood accumulation in the caudal vein, etc.

### Knockdown of *mvda* causes epidermal abnormalities

**Fig. 2:**
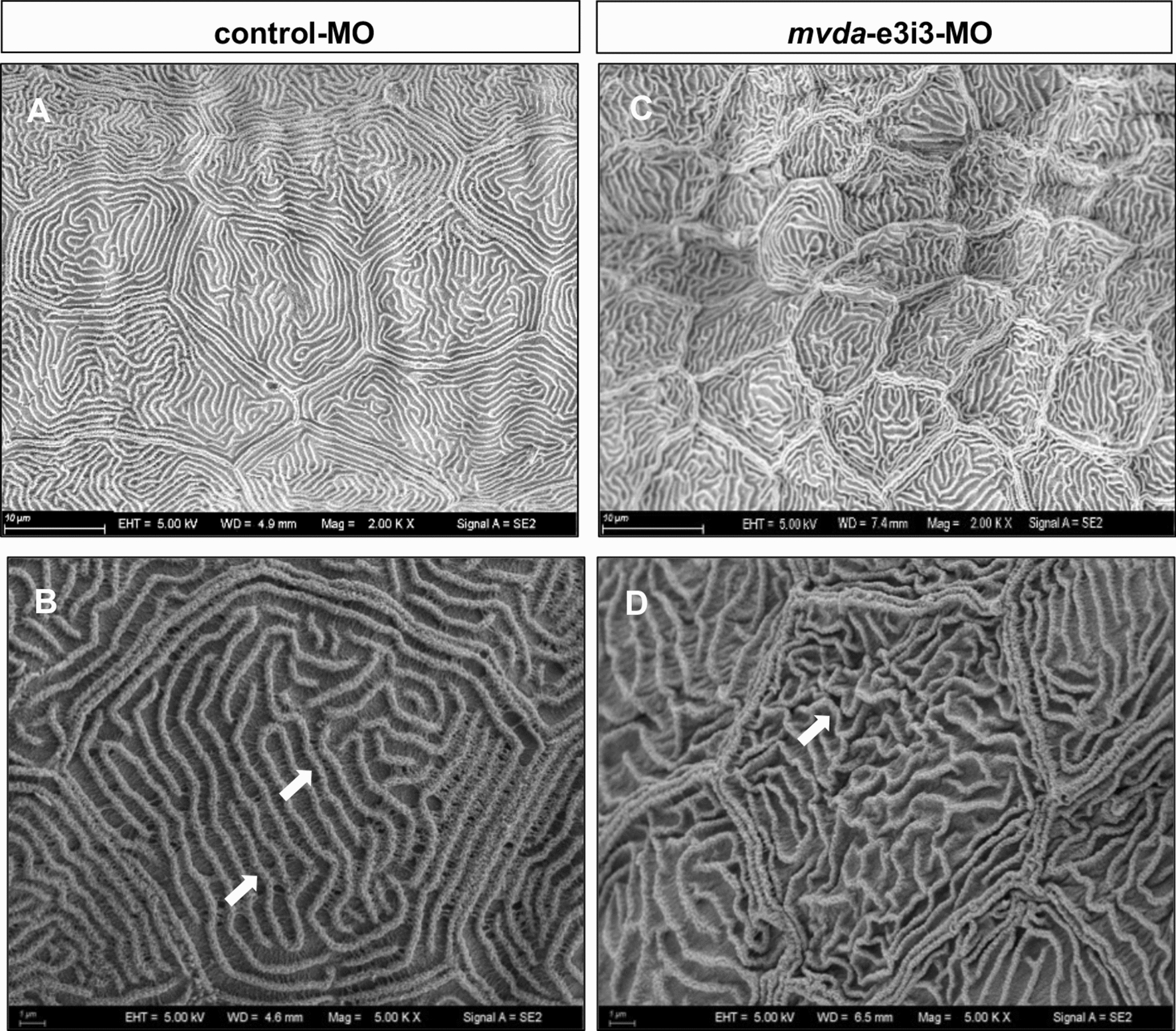
SEM of the skin surface in 4-dpf control fish and mvda morphants. **A**, **B** Control group revealed well-demarcated keratinocytes with regular arranged microridges (**B**, white arrows). **C**, **D** *mvda*-e3i3-MO-injected zebrafish revealed keratinocytes with shrunken borders and microridges. The microridges showed tortuous architecture and irregular intervals (**D**, white arrow). dpf, days post fertilization. Scale bars = 10 µm (**A**, **C**) and 1 µm (**B**, **D**)

**Fig. 3:**
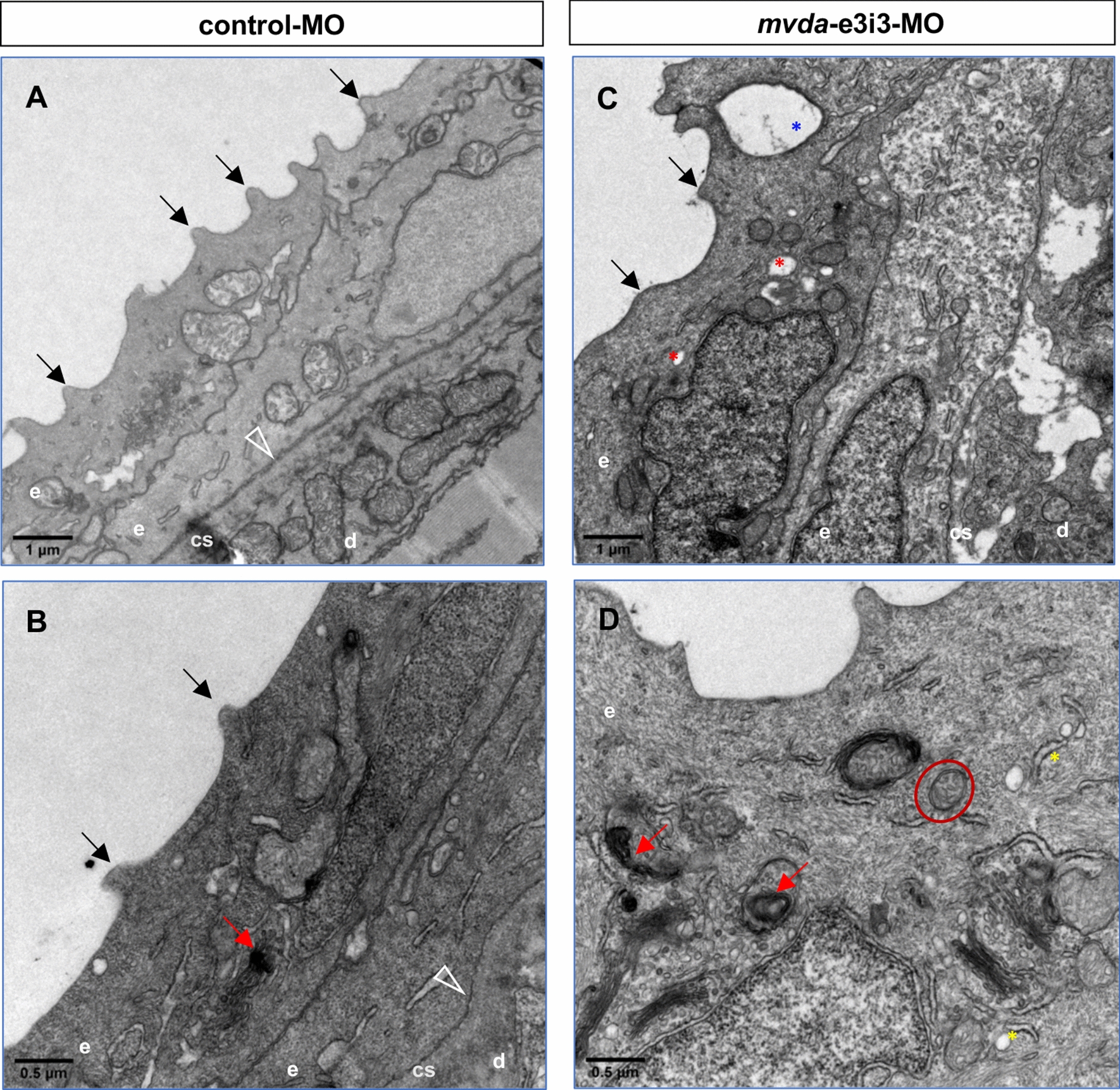
TEM of 4-dpf larvae injected with control or mvda morpholinos. **A**, **B** Keratinocytes of the control group revealed regular arranged spicules (black arrows) and cytoplasmic keratin filaments. **C**, **D** Keratinocytes of the *mvda*-e3i3-MO-injected zebrafish revealed disorganized spicules (black arrows), disordered cytoplasmic keratin filaments, distended rough endoplasmic reticulum (yellow asterisks), swollen mitochondria (red circle), autophagosomes and mitophagy (red arrowheads), vesicles (red asterisks), and rupture of cell–cell contacts (blue asterisk). Open arrowheads point to the basement membrane; e, epidermis; d, dermis; cs, collagenous stroma; dpf, days post fertilization. Scale bars = 1 µm (**A**, **C**) and 0.5 µm (**B**, **D**)

As indicated in Figs. 2 and 3, the epidermis of *mvda* morphants (4-dpf larvae) was compared with that of the control group using scanning electron microscopy (SEM) and transmission electron microscopy (TEM). The keratinocytes of *mvda* morphants revealed tortuous architecture and irregular intervals of the microridges, showing a disorganization of the spicules on the outer surface. The cytoplasmic keratin filaments were assembled either parallel to each other or at a right angle in the control group. However, these regular arrangements were altered in *mvda* morphants. Moreover, other abnormalities were observed, such as distended rough endoplasmic reticulum, swollen mitochondria, mitophagy, and rupture of cell–cell contacts.

### Loss of *mvda* induces vascular defects in vivo

**Fig. 4:**
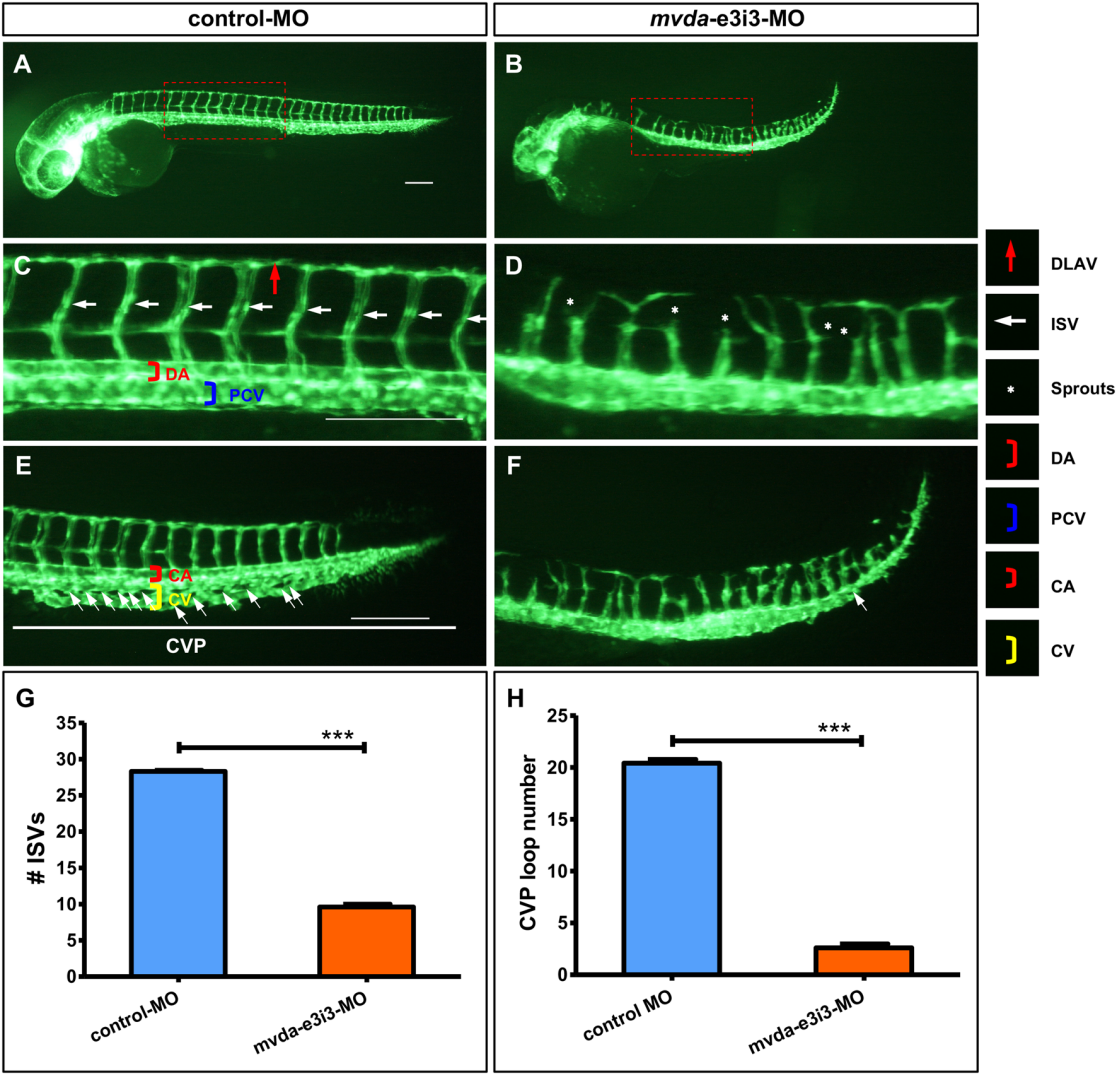
Morpholino knockdown of *mvda* impairs the trunk angiogenesis and CVP formation in zebrafish. **A**–**F** Representative fluorescent images of *Tg(fli1a:EGFP)*^*y1*^ embryos at 2-dpf, with the vascular structures visualized by eGFP fluorescence. The boxed regions of A and B are shown at a higher magnification in C and D, respectively. **C** ISVs and DLAV showed regular development in the embryos injected with control-MO. **D** Compared with the control group, embryos injected with *mvda*-e3i3-MO presented a lower number of incomplete ISVs and ectopic sprouts (asterisks) of DA. **E** In control embryos, CVP was formed honeycomb-like structures at the tail around 2-dpf (white arrows). **F** In contrast, *mvda* knockdown resulted in specific defects in CVP formation. **G** Quantification of the number of complete ISVs shows a significant decrease in *mvda* morphants. **H** Quantification of loop formation at CVP shows a 7.8-fold decrease in *mvda* morphants. Columns, mean; bars, SEM (n = 10; unpaired student’s t-test) ***P < 0.0001. *ISV* intersegmental vessel, *DLAV* dorsal longitudinal anastomotic vessels, *DA* dorsal aorta, *CVP* caudal vein plexus, *PCV* posterior cardinal vein, *CA* caudal artery, *CV* caudal vein, *dpf* days post fertilization. Scale bars = 100 µm

*Tg(fli1a:EGFP)*^*y1*^ zebrafish embryos, injected with control-MO, showed regular vascular structures with natural intersegmental vessels (ISVs) and dorsal longitudinal anastomotic vessels (DLAV) at 2-dpf (Fig. 4A, C). In contrast, those injected with *mvda*-e3i3-MO showed impaired trunk angiogenesis in zebrafish with a lower number of incomplete ISVs and ectopic sprouts of the dorsal aorta (DA) (Fig. 4B, D).

The caudal vein plexus (CVP) was formed as honeycomb-like structures at the tail around 2-dpf in the control embryos (Fig. 4E). However, *mvda*-deficient zebrafish exhibited a 7.8-fold decrease in the loop number of CVP formation at 2-dpf (Fig. 4F, H). These defects were consistent with the caudal vein blood accumulation phenotype of *mvda* morphants.

### Knockdown of *mvda* causes fin-reduction phenotypes and obvious apoptosis in the tail, heart, and eyes of zebrafish

**Fig. 5:**
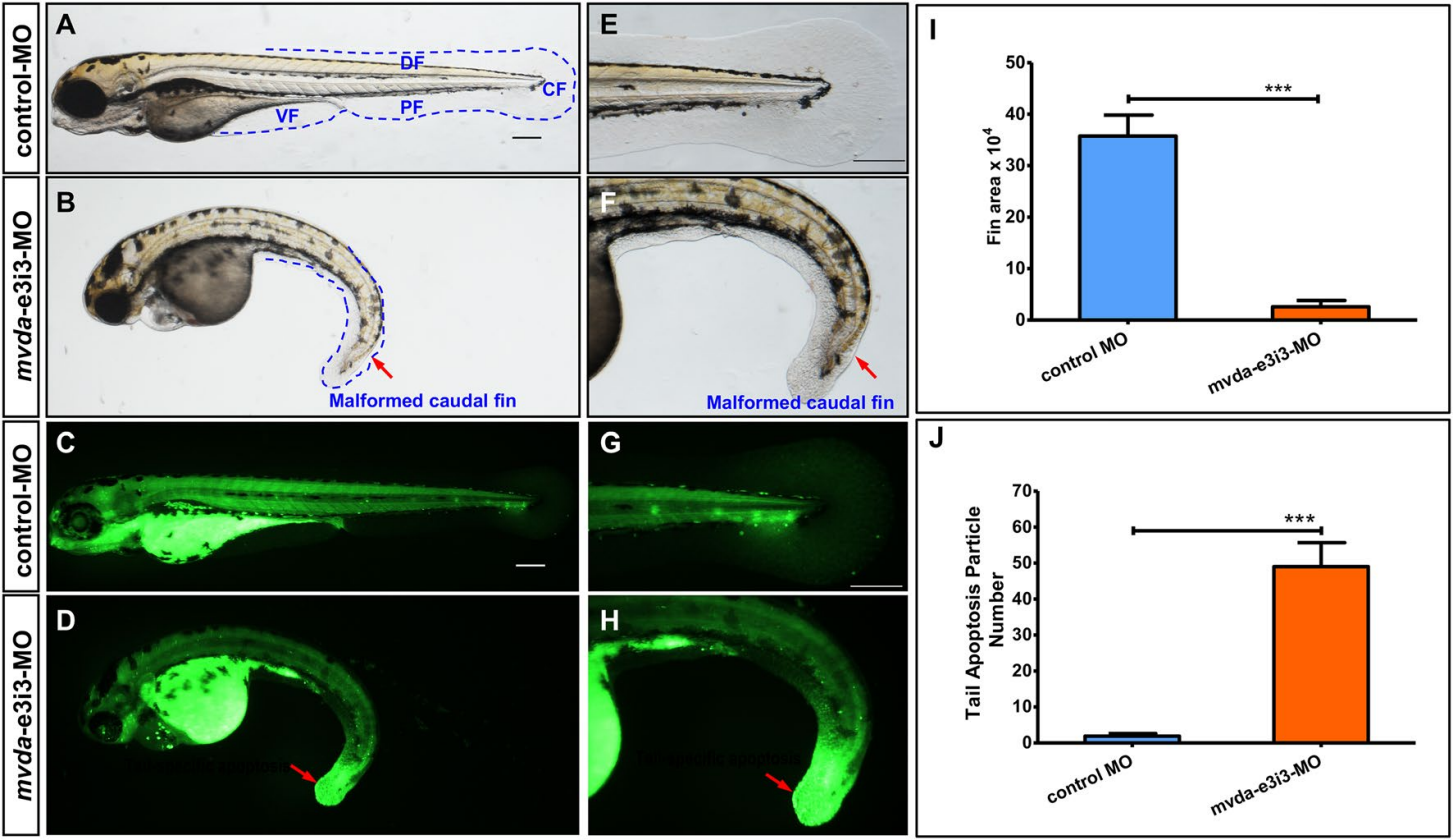
*mvda* deficiency induces fin-reduction phenotypes and tail-specific apoptosis. **A** normal fins, control-MO; **B** fin-reduction phenotypes after *mvda*-e3i3-MO injected; **E**–**F** caudal fin is shown at higher magnification. Dashed lines indicate the morphology of fins. Control-MO-injected embryos and embryos injected with *mvda*-e3i3-MO were stained with acridine orange at 4-dpf. Apoptotic cells are visible as bright green spots, and less bright homogenous green or black staining is unspecific background staining. **C**, **G** Control-MO-injected zebrafish exhibited few or no apoptotic cells in whole organism. **D**, **H** In contrast, significantly increased staining was observed throughout the tail in zebrafish injected with *mvda*-e3i3-MO (red arrows). **I** Quantification of area at fin shows a 13.9-fold decrease in *mvda* morphants. Error bars, s.e.m.; ***P < 0.0001(n = 10; ANOVA). **J** Quantification of apoptosis particle number at tail shows a 25.8-fold increase in *mvda* morphants. Error bars, s.e.m.; ***P < 0.0001(n = 10; Student’s t test); A–H: lateral view, anterior, left. *CF* caudal fin, *DF* dorsal fin, *PF* pelvic fin, *VF* ventral fin, *dpf* days post fertilization. Scale bars = 100 µm

**Fig. 6:**
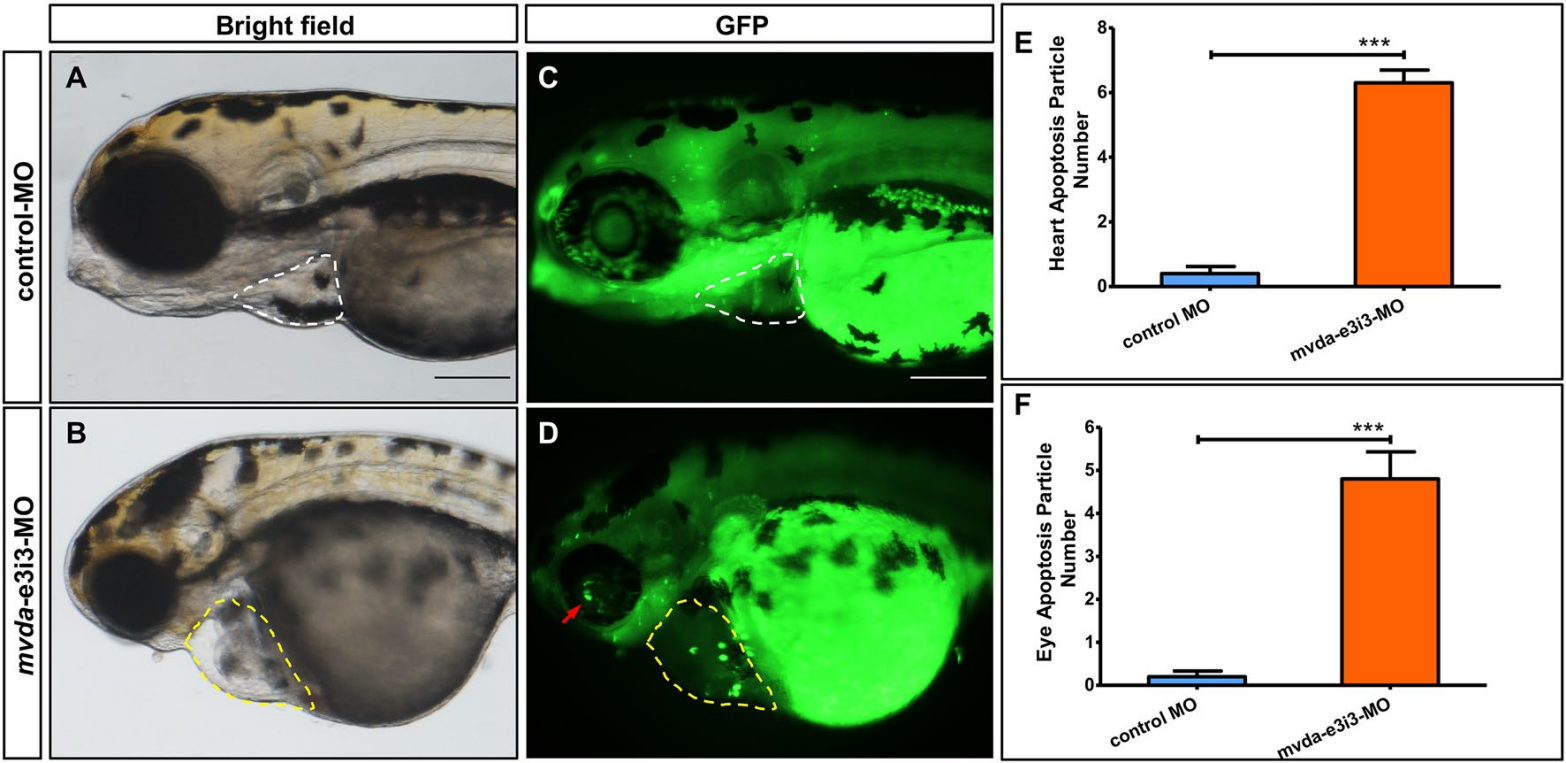
Morpholino knockdown of *mvda* induces potent apoptosis in heart and eyes. Control-MO-injected embryos and embryos injected with *mvda*-e3i3-MO were stained with acridine orange at 4-dpf. Apoptotic cells are visible as bright green spots, and less bright homogenous green staining is unspecific background staining. **A**, **C** Control-MO-injected zebrafish exhibited few or no apoptotic cells in heart and eyes. **B**, **D** In contrast, significantly increased staining was observed throughout the heart and eyes in zebrafish injected with *mvda*-e3i3-MO (circled areas and red arrow). **E** Quantification of apoptosis particle number at heart shows a 15.8-fold increase in *mvda* morphants. **F** Quantification of apoptosis particle number at eyes shows a 24-fold increase in *mvda* morphants. Error bars, s.e.m.; ***P < 0.0001(n = 10; Student’s t test); A–D: lateral view, anterior, left. *dpf* days post fertilization. Scale bars = 100 µm

Compared with normal fins in the control group (Fig. 5A, E), fin-reduction phenotypes were presented in *mvda*-e3i3-MO-injected zebrafish. The dorsal, ventral, pelvic, and caudal fins were found shrunk or absent (Fig. 5B, F), showing a 13.9-fold decrease in the fin area of *mvda* morphants (Fig. 5I). This indicated that *mvda* knockdown could induce antiproliferation. Acridine orange staining was used to detect the dying cells in vivo. Zebrafish injected with control-MO exhibited few or no apoptotic cells in the whole organism (Fig. 5C, G), whereas *mvda*-e3i3-MO-inected zebrafish showed significantly increased staining (Fig. 5D, H) and a 25.8-fold increase in apoptosis at the tail (Fig. 5J). Similarly, enhanced apoptosis was found in the heart and eyes of *mvda* morphants (Fig. 6).

## Discussion

The *mvda*-encoded enzyme catalyzes the sixth reaction of the mevalonate pathway, which exerts essential functions in multiple cellular processes regulating cell growth, differentiation, and proliferation [11, 12]. As expected, in *mvda*-e3i3-MO-injected zebrafish, anomalous microridges were observed on the contour of the epidermis. Furthermore, pathologically changed organelles, including distended rough endoplasmic reticulum, swollen mitochondria, and mitophagy, were found in the epidermal cells through SEM and TEM. These findings indicated the epidermal defects caused by *mvda* deficiency. Mitophagy is a form of autophagy that removes dysfunctional or superfluous mitochondria, thus maintaining the quality of mitochondria [13]. Several studies have shown that defective removal of damaged mitochondria would lead to hyperactivation of inflammatory signaling pathways because of abnormal mitochondria' immunogenic capabilities [14, 15]. Mitophagy and autophagy impairment, caused by impaired mevalonate pathway, might be responsible for the pathologically changed organelles observed in our study [16–18]. Moreover, a significant enhancement of apoptosis was found in the tail, heart, and eyes of *mvda* morphants. The decrease in protein prenylation levels not only induces autophagy impairment, but also triggers an increase of apoptosis by mitochondrial damage [19]. Previous study indicates that statins stimulate apoptotic cell death in various proliferating tumor cells in a cholesterol-lowering-independent manner [20]. Knockdown of *mvda* might have a similar effect to blocking the mevalonate pathway by statins, causing mitochondria damage and mitophagy, triggering an inflammatory response, and promoting cell death.

In this study, we found that knockdown of *mvda* could cause ectopic sprouts in trunk angiogenesis, pericardial oedema, blood accumulation in the caudal vein, and impaired formation of CVP in zebrafish, which together showed impaired vascular development. These observations supported the previous studies using 3-hydroxy-3-methylglutaryl coenzyme-A (HMG-CoA) reductase inhibitor to block the mevalonate pathway. [21–23]. *mvda* knockdown might lead to reduced activity of the mevalonate pathway, which proper function is proposed to be essential for regular endothelial cell migration, heart-tube formation, and angiogenesis in zebrafish. The vascular endothelial growth factor (VEGF) signaling cascade has been linked to multiple steps of vascular endothelial cell development, playing a known role in the survival of vascular endothelial cells and the formation of vasculature during embryogenesis [24, 25]. Loss of vasculature, pericardial oedema, and blood accumulation phenotypes were observed in *VEGF-A* morphants, revealing the fundamental character of *VEGF-A* in angiogenesis [26]. Since *mvda* gene knockdown resulted in impaired vascular development, whether VEGF signaling was consequently altered remains to be clarified.

As it is known to all, the appropriate vasculature maintains an adequate supply of blood and oxygen, which is required for the development and survival of organs, including the epidermis. Abnormal angiogenesis is ineffective in establishing blood flow and inevitably results in defects of development [27]. Our results implied that the non-functional vascular sprouts of zebrafish, injected with *mvda*-e3i3-MO, could potentially induce hypoxia and nutritional deficiency, leading to the abnormal morphology, mitophagy, and apoptosis. Low oxygen levels might trigger an inflammatory response and be involved in the cellular mechanisms associated with mitochondrial damage. Moreover, hypoxia might stimulate the transcription of the *VEGF* gene, resulting in increased angiogenesis and proliferation in the epidermis [27]. Of note, irregular vessels are observed in PK lesions through dermoscopic examination [28–30], which are usually neglected by naked eyes. In addition, one case of primary cardiac amyloidosis associated with PK was reported in the literature [31]*.* Based on these observations, we inferred that abnormal angiogenesis might be the primary effect of *mvda* deficiency.

The mvda-deficient zebrafish was generated to study the biological function of gene. MOs and gene-editing mutagenesis are very different technologies and, even if both work perfectly, may yield different results on targeting the same gene. Gene-editing technologies work on DNA, are permanent, and often require rounds of backcrossing to stabilize, allowing time for physiological, epigenetic, or genetic change. MOs working on RNA, can attenuate targets that may be lethal when knocked out with gene editing, and cause transient and concentration dependent knockdowns with assessment of outcome usually done within 5 days. We suggest that different reported outcomes of MOs and gene editing may be due to the differences in timing and site of action [32]*.* To our knowledge, *MVD* mutations in PK patients are heterozygous, where one functional allele is still present. Even though *mvda*-deficient zebrafish could not accurately mimic the phenotype of PK, the epidermal abnormalities in *mvda* morphants might signify the pathological characteristics of that in PK lesions, such as premature apoptosis [33]. Further studies are necessary to elucidate the underlying molecular mechanism.

In addition, it is known that tissue injury causes vascular leakage and the influx of blood, which induces the acute inflammatory. Various proteases are required for the efficient migration of macrophages to the injury site in the zebrafish model [34]. However, our data showed that knockdown of *mvda* didn’t inhibit macrophage recruitment in response to acute injury (Additional file 1: Figure S1). *mvda* seems not to be involved in the macrophage-mediated innate inflammatory response. The major limitation of this study was that we didn’t perform rescue experiments and co-injection of *tp53*-MO due to a limited budget and time. Therefore, the apoptosis resulted from *mvda* deficiency should be interpreted carefully.

## Conclusions

Taken together, these results showed the pathological alterations in developing zebrafish caused by *mvda* knockdown. Thus, *mvda* is required for the early development of zebrafish. Our findings might contribute to understanding the biological function of the human *MVD* gene.

## Materials and methods

### Zebrafish care and maintenance

Adult zebrafish were maintained at 28.5 °C on a 14 h light/10 h dark cycle [35]*.* Five to six pairs of zebrafish were set up for nature mating every time. On average, 200–300 embryos were generated. Embryos were maintained at 28.5 °C in fish water (0.2% Instant Ocean Salt in deionized water). The embryos were washed and staged according to standard procedures [36]. The establishment and characterization of the *Tg(fli1a:EGFP)*^*y1*^ transgenic line has been described elsewhere [37]. The zebrafish facility at SMOC (Shanghai Model Organisms Center, Inc.) is accredited by the Association for Assessment and Accreditation of Laboratory Animal Care (AAALAC) International.

### Zebrafish microinjections

Gene Tools, LLC (http://www.gene-tools.com/) designed the morpholino. Antisense MO (GeneTools) was microinjected into fertilized one-cell stage embryos according to standard protocols [38]. The sequence of the exon 3-intron 3 splice-blocking *mvda* morpholino (*mvda*-e3i3-MO) was 5ʹ-TATGAAAGCCAGGAACATACTTTCC-3ʹ, and the sequence for the standard control morpholino was 5ʹ-CCTCTTACCTCAGTTACAATTTATA-3ʹ (Gene Tools). For *mvda* gene knockdown experiment, 4 ng of control-MO or *mvda*-e3i3-MO was used per injection. Primers spanning *mvda* exon 1 (forward primer: 5ʹ-ACTGGGGTAAACGTGATGAAGAT-3ʹ) and exon 4 (reverse primer: 5ʹ-GCCAGGCCAGCAGCAGT-3ʹ) were used for RT-PCR analysis for confirmation of the efficacy of the *mvda*-e3i3-MO. The primer *ef1α* sequences used as the internal control were 5ʹ-GGAAATTCGAGACCAGCAAATAC-3ʹ (forward) and 5ʹ-GATACCAGCCTCAAACTCACC-3ʹ (reverse).

### Quantitative real-time PCR

Total RNA was extracted from 30 to 50 embryos per group in Trizol (Roche) according to the manufacturer's instructions. RNA was reverse transcribed using the the PrimeScript RT reagent Kit with gDNA Eraser (Takara). Quantification of gene expression was performed in triplicates using Bio-rad iQ SYBR Green Supermix (Bio-rad) with detection on the Realplex system (Eppendorf). Relative gene expression quantification was based on the comparative threshold cycle method (2 − ΔΔCt) using *ef1α* as endogenous control gene. Primer sequences were shown in Additional file 4: Table S1.

### Scanning electron microscopy (SEM)

Zebrafish were fixed in 4% paraformaldehyde and 2.5% glutaraldehyde at 4℃ overnight. Specimens were then rinsed in 0.1 M phosphoric acid solution, postfixed in 1% OsO4 overnight, rinsed again in 0.1 M phosphoric acid solution then dehydrated in a graded series of ethanol dilutions to 100% ethanol. Infiltration was done by first 1:1 ethonal: acetone for 15 min, and finally pure acetone for 30 min. Specimens were then critically dried using liquid CO2 as the substitution medium by HCP-2 critical point dryer (Hitachi, Japan), then adhered to aluminum specimen mounts using carbon conductive cement. A versatile sputter coater/turbo evaporator (Quorum Q150T, United Kingdom) was used to gold coat the samples. Imaging was carried out using a field-emission scanning electron microscopy (Zeiss Gemini 300, Germany).

### Transmission electron microscopy (TEM)

Zebrafish were fixed in 2.5% glutaraldehyde in 0.1 M cacodylate buffer (pH 7.4) for 2 h at room temperature. Specimen were postfixed in 1% OsO4 in 0.1 M cacodylate buffer and then dehydrated by ethanol through the following steps: 30% 10 min, 50% 10 min, 70% overnight, 80% 20 min, 90% 20 min, three times in 100% ethanol 20 min/each, and then two times in acetone 30 min/each. Infiltration was done by first 1:1 resin: acetone for 1 h, 2:1 resin: acetone for 1 h, pure resin for 1 h, and finally pure resin overnight. The fishes were embedded in an 70 °C oven overnight. The 70% ethanol was saturated with uranylacetate for contrast enhancement. The specimens were embedded in EMBED-812 EMBEDDING KIT (Electron Microscopy Sciences). Ultrathin sections (70–80 nm) were cut on an Ultracut ultramicrotome (Leica UC6, Germany), mounted on pioloform-coated 50 mesh grids, and contrasted with lead citrate for 5 min. Ultrathin sections and replicas were observed and examined with electron microscope (JEOL-1230).

### Zebrafish angiogenesis study

To evaluate blood vessels formation in zebrafish, fertilized one-cell *Tg(fli1a:EGFP)*^*y1*^ transgenic line embryos were injected with 4 ng *mvda*-e3i3-MO. At 2-dpf, embryos were dechorionated, anesthetized with 0.016% MS-222 (tricaine). Zebrafish were then oriented on lateral side (anterior, left; posterior, right; dorsal, top), and mounted with 3% methylcellulose in a depression slide for observation by fluorescence microscopy. The morphology of the caudal vein plexus and the number of intersegmental vessels that connect the dorsal aorta to the dorsal longitudinal anastomotic vessels were analyzed.

### Acridine orange staining for apoptosis

Control-MO-injected embryos and embryos injected with *mvda*-e3i3-MO (4 ng of control-MO or *mvda*-e3i3-MO was used per injection) were immersed in 5 μg/ml AO (acridinium chloride hemi-[zinc chloride], Sigma-Aldrich) in fish water for 60 min at 4-dpf [39, 40]. Next, zebrafish were rinsed thoroughly in fish water three times (5 min/wash) and anaesthetized with 0.016% MS-222 (tricaine methanesulfonate, Sigma-Aldrich, St. Louis, MO). Zebrafish were then oriented on their lateral side and mounted with 3% methylcellulose in a depression slide for observation by fluorescence microscopy.

### Image acquisition

Embryos and larvae were analyzed with Nikon SMZ 1500 Fluorescence microscope and subsequently photographed with digital cameras. A subset of images was adjusted for levels, brightness, contrast, hue, and saturation with Adobe Photoshop 7.0 software (Adobe, San Jose, California) to optimally visualize the expression patterns. Quantitative image analyses processed using image based morphometric analysis (NIS-Elements D3.1, Japan) and ImageJ software (U.S. National Institutes of Health, Bethesda, MD, USA; http://rsbweb.nih.gov/ij/). Positive signals were defined by particle number using ImageJ. 10 animals for each treatment were quantified and the total signal per animal was averaged.

### Statistical analysis

All data are presented as mean ± SEM. Statistical analysis and graphical representation of the data were performed using GraphPad Prism 5.0 (GraphPad Software, San Diego, CA). Statistical significance was performed using a Student’s t test or χ2 test as appropriate. Statistical significance is indicated by *, where P < 0.05, and ***, where P < 0.0001.

## Supplementary Information

**Additional file 1: Figure S1.** *mvda* knockdown presents normal macrophage chemotaxis in zebrafish model *Tg(zlyz:EGFP)*. The materials and method of zebrafish in vivo macrophage migration assays and the resulting figure.

**Additional file 2: Video S1.** Comparison of heartbeat and circulation in the caudal vein between the control group and *mvda* morphants. Heartbeat and circulation in the caudal vein were visible in the control fish but were abnormal in *mvda* morphants at 2-dpf.

**Additional file 3: Video S2.** Comparison of heartbeat and circulation in the caudal vein between the control group and *mvda* morphants. Heartbeat and circulation in the caudal vein were visible in the control fish but were abnormal in *mvda* morphants at 2-dpf.

**Additional file 4: Table S1.** qRT-PCR primers for zebrafish. The primer sequences for qRT-PCR.

## Data Availability

All data generated or analyzed during this study are included in this published article.
