# Supplementary material for: Mvda is required for zebrafish early development
Source: Biol Res. 2021 May 29;54:17. doi: 10.1186/s40659-021-00341-7 (PMC8164810; doi:10.1186/s40659-021-00341-7)
Supplement: Supplementary file 1 — Additional file 1: Figure S1. mvda knockdown presents normal macrophage chemotaxis in zebrafish model Tg(zlyz:EGFP). The materials and method of zebrafish in vivo macrophage migration assays and the resulting figure. [file 40659_2021_341_MOESM1_ESM.docx]

**Zebrafish *in vivo* macrophage migration assays**

To evaluate macrophage migration in zebrafish, fertilized one-cell *Tg(zlyz:EGFP)* transgenic line embryos were injected with 4ng *mvda*-e3i3-MO or control-MO. Zebrafish *Tg(zlyz:EGFP)* larvae (at 3-dpf) were anaesthetised with Tricaine (MS-222) and their tailfins amputated using sharp needles to induce the migration of macrophage. After injury, the zebrafish larvae were transferred to fresh extract solution for 24 hours before visual inspection using a fluorescence stereomicroscope. At 24 hours post tail transection (hptt), larvae were anesthetized with 0.016% MS-222 (tricaine) and the number of macrophages recruited to the wound site was counted.
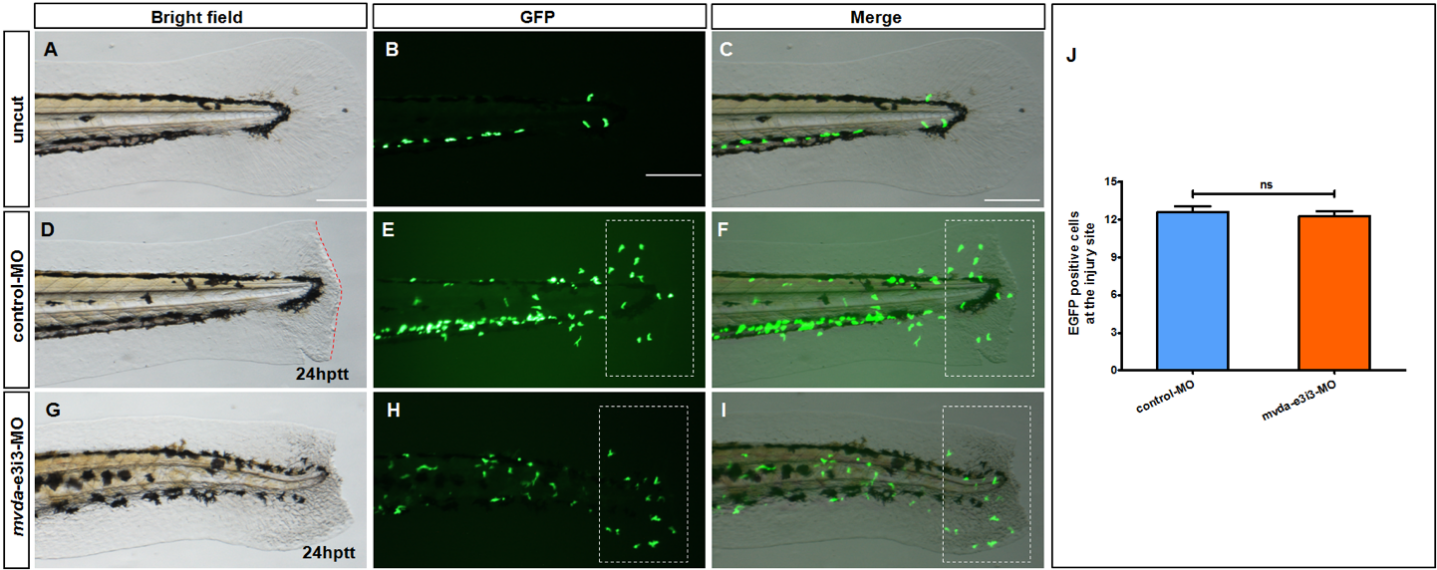


**Figure S1.** ***mvda* knockdown present normal macrophage chemotaxis in zebrafish model *Tg(zlyz:EGFP)*.**

(**A-C**) Macrophages were quiescent on an uninjured 4-dpf larva. (**D-F**) Macrophage migration in response to acute injury is visualized in zebrafish model *Tg(zlyz:EGFP)*. *Tg(zlyz:EGFP)* embryos (at 3-dpf) were subjected with tail fin transection for generating zebrafish macrophage migration model. The red dotted line indicates the incision of tail transection, and the square region means the highly inflamed region. (**G-I)** Compared with control fish, embryos injected with *mvda*-e3i3-MO presented no significant change in macrophage migration. (**J**) Quantification of the number of macrophages that migrated to the highly inflamed regions (white boxes) in *Tg(zlyz:EGFP)* embryos with tail transection at 24-hptt. Columns, mean; bars, SEM (n =10; Student’s t test; ns, not significant). hptt, hour post tail transection; dpf, days post fertilization. Scale bars=100 µm.
